# Effect of prenatal ambient temperature on the performance physiological parameters, and oxidative metabolism of Japanese quail (*Coturnix coturnix japonica*) layers exposed to heat stress during growth

**DOI:** 10.1038/s41598-021-89306-0

**Published:** 2021-05-07

**Authors:** Thaís Pacheco Santana, Eliane Gasparino, Angélica de Souza Khatlab, Claudson Oliveira Brito, Leandro Teixeira Barbosa, Susan J. Lamont, Ana Paula Del Vesco

**Affiliations:** 1 Department of Animal Science, Federal University of Sergipe, São Cristóvão, Brazil; 2 Department of Animal Science, State University of Maringá, Maringá, Brazil; 3 Department of Animal Science, Iowa State University, Ames, USA

**Keywords:** Biochemistry, Genetics, Molecular biology

## Abstract

A strategy to mitigate the negative effects of stress on animals is to enhance their ability to beneficially respond to stressful conditions. This study aimed to assess whether prenatal ambient temperature influences the response of Japanese quail (*Coturnix coturnix japonica*) chicks to environmental challenges during growth. The experiment was conducted in a 2 × 2 factorial arrangement: two temperature conditions for the mothers (thermoneutral and heat stress by continuous exposure to 32 °C) and two offspring ambient temperature conditions (thermoneutral and heat stress by intermittent exposure to 34 °C for 6 h/day from 15 to 35 days of age). Heat stress in mothers led to lower laying rate, egg mass, expression of methionine sulfoxide reductase A (*MSRA*) gene, and antioxidant capacity as well as higher chick mortality rate (1–15 days of age). Maternal heat stress led to lower weight gain and total antioxidant capacity and higher feed conversion ratio. Maternal temperature × Offspring temperature interaction effects were observed on carbonylated protein content and *HSP70*, *GSS*, and *MSRA* gene expression. It was observed that, for chicks hatched from heat-stressed mothers, exposure to heat stress led to higher carbonylated protein content and *HSP70* expression than exposure to thermoneutral conditions. Maternal heat stress was also responsible for increasing *GSS* expression in chicks grown under thermoneutral conditions. Chicks hatched from non-stressed mothers and subjected to heat stress had higher *MSRA* expression compared to chicks maintained in a thermoneutral environment. Our results show that, although maternal heat stress had no negative effects on performance or oxidative metabolism of offspring grown under thermoneutral conditions, it was associated with lower performance and higher protein oxidation in offspring exposed to heat stress during growth. These results could be due in part to alterations in the expression of genes related to antioxidant capacity.

## Introduction

Temperature above optimal ranges is one of the greatest challenges in animal production^1,2^. When birds are subjected to high environmental temperatures, behavioral and physiological mechanisms are activated in an attempt to reduce metabolic heat production and increase heat dissipation. When these mechanisms are not sufficient, there is an imbalance between the amount of heat produced or gained and the amount of heat lost to the environment, and a heat stress (HS) condition takes place^3^.

Some of the effects of heat stress are increased chemical and biochemical reaction rates along with increased body temperature and reactive oxygen species (ROS) generation^4^. In addition, heat stress can reduce triiodothyronine concentration, resulting in reduced protein deposition through protein turnover in birds. Heat stress is also related to alterations in the activity of the neuroendocrine system of poultry, resulting in activation of the hypothalamic–pituitary–adrenal (HPA) axis and increased plasma corticosterone concentration^5^. The immune capacity is also affected, making animals more susceptible to diseases^6^. Due to the above-mentioned alterations, heat stress can exert negative effects on animal performance by reducing feed intake, weight gain, and feed efficiency^7^. In bird breeders, heat stress can also decrease the reproductive capacity^8^ by reducing the synthesis of the gonadotropins follicle-stimulating hormone and luteinizing hormone as well as estradiol, which results in hormonal dysfunction associated with low follicle dominance, low yolk quality and egg production, low fertility rate and hatchability, and ultimately a lower number of live hatchlings^7–9^.

Chick development may be affected by the maternal environment^10–12^. It is known that the environment can influence gene expression through epigenetic mechanisms and the resulting phenotypic alterations can be passed from parent to offspring through intergenerational and transgenerational epigenetic inheritance^13,14^. Therefore, the conditions experienced by mothers can either prepare offspring to respond better to stressful conditions during growth or produce undesirable effects for animal production^12,15^. Duo to the importance of this topic, there is a growing effort in the scientific community to study how parental environment can affect offspring development. In a comprehensive study, Videla et al.^16^ assessed whether feed supplementation with thymol could positively modulate the immune response of adult quails when exposed to simultaneous environmental challenges: chronic heat stress exposure and inoculation with inactivated *Salmonella* Enteritidis. In addition, it was also studied the transgenerational effects of the experienced situations on immune representative variables on their eggs and offspring. The results showed that for the chicks whose parents were exposed to chronic heat stress respectively showed a higher inflammatory response and lower titers than the chicks whose parents were not exposed to HS. According to the authors, these findings are consistent with a parental generation programming of their chicks, and it would be reasonable to question what the results would be whether the offspring were to be exposed to the same environmental conditions than the parental birds.

Thus, in the present study we hypothesized that heat stress in the parental generation could influence heat stress coping capacities in their offspring. To assess our hypothesis, we had as main objective to evaluate the performance, physiological indicators and oxidative metabolism of chicks hatched from heat-stressed or non-stressed mothers and raised under thermoneutral conditions or heat stress during the growth phase.

## Results

### Effect of ambient temperature on mothers performance and oxidative metabolism

Mothers subjected to heat stress presented higher body temperature than mothers under thermoneutral condition (42.70 vs. 41.25 °C, respectively, *P* < 0.05). The effects of ambient temperature on mothers performance are presented in Table 1. As expected, mothers exposed to heat stress had a lower laying rate (about 28% lower) and egg mass than mothers under thermoneutral conditions (*P* < 0.05). However, ambient temperature did not affect egg weight, hatchability of total and fertile eggs, or hatchling weight at birth. No effects of high temperature treatment were observed on number and weight of mature follicles or on the relative weights of the ovary, liver, heart, and spleen.

**Table 1:** Performance of the mothers. Female quail hens (*n* = 40) were divided and subjected to two environmental conditions: thermoneutral (23 °C and 60% relative humidity) (*n* = 10) and heat stress (continuous exposure to 32 °C and 60% relative humidity) (*n* = 30).

Parameter	Environmental conditions		SE	*P* value
	Thermoneutral	Heat stress		
Initial body weight (g)	160.00	165.83	4.05	0.5307
Final body weight (g)	173.33	171.67	1.30	0.7402
Feed intake (g)	850.00	625.17	51.65	0.0487*
Feed conversion ratio (g feed/g egg mass)	2.74	2.77	0.23	0.9142
Feed conversion ratio per dozen eggs (kg feed/g dozen eggs)	2.48	2.56	0.19	0.7727
Total number of eggs produced	28.66	20.17	1.08	0.0002*
Egg laying rate (%)	95.55	67.22	2.60	0.0002*
Egg weight (g)	10.93	11.17	0.32	0.6034
Egg mass (g)	313.61	224.53	10.37	0.0008*
Hatchability (%)	68.51	62.47	5.34	0.5750
Hatchability of fertile eggs (%)	71.69	75.59	6.11	0.7061
Hatchling weight at birth (g)	7.72	7.98	0.15	0.2174
Number of mature follicles	4.20	4.10	0.31	1.0000
Weight of mature follicles	1.32	1.13	0.13	0.3325
Relative weight of ovary (%)	3.66	3.45	0.30	0.6491
Relative weight of liver (%)	2.82	3.03	0.17	0.4135
Relative weight of heart (%)	0.81	0.85	0.01	0.3497
Relative weight of spleen (%)	0.05	0.04	0.01	0.4748

*SE* standard error.

An asterisk (*) indicates a significant difference by the *t* test (*P* < 0.05).

Table 2 shows the results for gene expression and antioxidant parameters in the liver of the mothers. We observed that *MSRA* expression and total antioxidant capacity were lower in mothers subjected to heat stress. Ambient temperature did not influence *GPX7*, *GSS*, or *HSP70* expression or other oxidative metabolism parameters (*P* > 0.05).

**Table 2:** Effect of ambient temperature on the expression of heat-shock protein 70 kDa (*HSP70*), glutathione peroxidase 7 (*GPX7*), glutathione synthetase (*GSS*), and methionine sulfoxide reductase A (*MSRA*) genes, total antioxidant capacity, lipid peroxidation (TBARS), carbonylated protein, and heterophil/lymphocyte (H/L) ratio in the liver of the mothers.

Parameter	Environmental conditions				*P* value
	Thermoneutral		Heat stress		
	Mean	SE	Mean	SE	
*HSP70* (AU)	0.0177	0.0018	0.0152	0.0013	0.2741
*GPX7* (AU)	0.0045	0.0004	0.0045	0.0008	0.2446
*GSS* (AU)	0.0014	0.0003	0.0009	0.0002	0.2435
*MSRA* (AU)	0.2944^a^	0.0445	0.1115^b^	0.0128	0.0005
Total antioxidant capacity (%)	44.5431^a^	1.5408	37.3363^b^	1.1709	0.0549
TBARS (nmol TBARS/mg protein)	1.1077	0.0721	0.8398	0.1048	0.0614
Carbonylated protein (nmol carbonyl group/mg protein)	6.8028	0.1916	6.3844	0.1492	0.1156
H/L ratio	0.9153	0.0972	1.010	0.1478	0.6058

Female quail hens were raised in a thermoneutral environment (23 °C and 60% relative humidity) (*n* = 6) or subjected to heat stress (continuous exposure to 32 °C and 60% relative humidity) (*n* = 6). The 2^−∆CT^ method was used for relative quantification of gene expression, and results are expressed as arbitrary units (AU), which represent the expression of each gene normalized to the expression of β-actin mRNA. The results are presented as mean and standard error (SE).

^a,b^Different superscript letters in the same row indicate a significant difference by the *t* test (*P* < 0.05).

### Effect of maternal environment on early offspring performance (1–15 days of age)

For the assessment of the effect of maternal environment on the early stages of progeny development, all chicks were raised under the same conditions until 15 days of age. The mortality rate was lower among offspring of mothers kept under thermoneutral than under heat stress conditions (17.94 vs. 24.13%, *P* < 0.0010); however, weight at 15 days of age (41.03 vs. 42.27 g, *P* > 0.05) and weight gain (33.21 vs. 34.29 g, *P* > 0.05) were not affected by maternal environment.

### Effect of maternal environment on offspring performance and oxidative metabolism during growth (15–35 days of age)

At 15 days of age, chicks were divided and subjected to either thermoneutral or heat stress conditions. To assess how the maternal environment affects offspring response to ambient temperature, we assessed the interaction between mother temperature and offspring temperature on the performance and oxidative metabolism of chicks during the growth phase. When the interaction effect is not significant, main effects are presented.

There was no interaction effect on performance parameters (*P* > 0.05). However, there was a significant effect of maternal temperature on chick weight at 35 days of age (W35, *P* = 0.0099) and weight gain (WG, *P* = 0.0056), with chicks from mothers subjected to heat stress having lower W35 and WG than chicks from mothers kept under thermoneutral conditions. It was also observed a significant effect of offspring temperature on W35 (*P* = 0.0393), WG (*P* = 0.0109), and feed conversion ratio (FCR, *P* = 0.0478): chicks raised in heat stress had lower W35 and WG and higher FCR than thermoneutral chicks (*P* < 0.05). There was no effect of treatments on weight at 15 days of age or feed intake (Table 3).

**Table 3:** Performance of Japanese quail (*Coturnix coturnix japonica*) chicks raised under thermoneutral or heat stress conditions (*n* = 25).

	W15 (g)		W35 (g)		WG (g)		FI (g)		FCR (g/g)	
	Mean	SE	Mean	SE	Mean	SE	Mean	SE	Mean	SE
**Mothers TN**										
Offspring TN	40.53	2.06	132.47	2.59	91.93	1.99	369.86	33.89	4.16	0.40
Offspring HS	42.08	1.25	127.62	3.43	85.54	3.86	435.33	31.80	5.27	0.54
**Mothers HS**										
Offspring TN	41.11	2.12	125.83	3.50	84.72	4.04	384.24	40.97	4.54	0.59
Offspring HS	43.11	1.79	117.46	2.09	74.35	1.74	421.52	38.48	5.54	0.50
***Main effects***										
**Mothers**										
TN	41.25	1.24	130.21^a^	2.12	88.96^a^	2.13	400.26	23.81	4.68	0.34
HS	42.11	1.37	121.65^b^	2.22	79.53^b^	2.48	402.88	27.64	5.04	0.39
**Offspring**										
TN	40.75	1.49	129.98^a^	2.14	89.23^a^	2.04	375.25	25.65	4.31^b^	0.33
HS	42.50	1.02	123.46^b^	2.42	80.96^b^	2.64	429.68	23.97	5.38^a^	0.37
***P value***										
Mothers	0.6775		0.0099		0.0056		0.9939		0.5305	
Offspring	0.3624		0.0393		0.0109		0.1715		0.0478	
Mothers × Offspring	0.9062		0.5736		0.5305		0.7045		0.9166	

Results are presented as mean and standard error (SE).

*W15* chick weight at 15 days of age, *W35* chick weight at 35 days of age, *WG* weight gain, *FI* feed intake, *FCR* feed conversion ratio, *TN* thermoneutral, *HS* heat stress.

^a,b^Different subscript letters in the same column indicate a significant difference by the *t* test (*P* < 0.05).

Table 4 presents the results of oxidative parameters and H/L ratio. It was observed a significant effect of mother temperature × offspring temperature on carbonylated protein content (*P* = 0.0395). For chicks hatched from heat-stressed mothers, a heat-stress environment resulted in higher carbonylated protein content than a thermoneutral environment. There was no effect of offspring temperature for chicks hatched from non-stressed mothers.

**Table 4:** Oxidative parameters and H/L ratio of Japanese quail (*Coturnix coturnix japonica*) chicks raised under thermoneutral or heat stress conditions (*n* = 6).

	TBARS		Carbonylated protein		Total antioxidant capacity		H/L ratio	
	Mean	SE	Mean	SE	Mean	SE	Mean	SE
**Mothers TN**								
Offspring TN	1.31	0.06	7.75^b^	0.53	63.54	2.00	0.60	0.09
Offspring HS	1.44	0.11	7.85^ab^	0.71	62.87	3.38	0.46	0.13
**Mothers HS**								
Offspring TN	1.34	0.05	6.68^b^	0.82	57.07	3.38	0.48	0.11
Offspring HS	1.48	0.19	9.53^a^	0.23	54.27	0.91	0.40	0.01
***Main effects***								
**Mothers**								
TN	1.37	0.06	7.80	0.42	63.23^a^	1.50	0.53	0.08
HS	1.41	0.10	8.10	0.62	55.67^b^	1.71	0.44	0.05
**Offspring**								
TN	1.32	0.08	7.30	1.98	60.84	0.47	0.55	0.07
HS	1.46	0.06	8.62	1.90	58.96	0.47	0.43	0.07
***P value***								
Mothers	0.7708		0.6318		0.0047		0.3036	
Offspring	0.2143		0.0282		0.4695		0.4042	
Mothers × Offspring	0.9592		0.0395		0.6548		0.7952	

Results are presented as mean and standard error (SE).

*TBARS* lipid peroxidation, *H/L ratio* heterophil/lymphocyte ratio, *TN* thermoneutral, *HS* heat stress.

^a,b^Different superscript letters in the same column indicate a significant difference by Tukey’s test and the *t* test (*P* < 0.05).

A significant effect of mother temperature was observed on total antioxidant capacity. Chicks hatched from heat-stressed mothers had lower total antioxidant capacity than chicks hatched from mothers under thermoneutral conditions (63.23 vs. 55.67%, *P* = 0.0047). There was no treatment effect on H/L ratio (*P* > 0.05).

The results of gene expression are presented in Table 5. There was a mother temperature × offspring temperature interaction effect on *GSS* (*P* = 0.0101), *HSP70* (*P* = 0.0452), and *MSRA* (*P* = 0.0109) gene expression.

**Table 5:** Gene expression of Japanese quail (*Coturnix coturnix japonica*) chicks raised under thermoneutral or heat stress conditions (*n* = 6).

	*GPX7* (AU)		*GSS* (AU)		*HSP70* (AU)		*MSRA* (AU)	
	Mean	SE	Mean	SE	Mean	SE	Mean	SE
**Mothers TN**								
Offspring TN	0.03	0.01	0.01^b^	0.01	0.12^b^	0.04	0.30^b^	0.06
Offspring HS	0.06	0.02	0.03^a^	0.01	0.32^b^	0.11	0.59^a^	0.08
**Mothers HS**								
Offspring TN	0.05	0.01	0.02^a^	0.01	0.04^c^	0.01	0.46^ab^	0.07
Offspring HS	0.07	0.04	0.01^b^	0.01	0.54^a^	0.10	0.39^b^	0.06
***Main effects***								
**Mothers**								
TN	0.05	0.01	0.02	0.01	0.22	0.06	0.44	0.06
HS	0.06	0.02	0.02	0.01	0.26	0.07	0.43	0.04
**Offspring**								
TN	0.04	0.01	0.01	0.01	0.08	0.02	0.38	0.05
HS	0.07	0.02	0.02	0.01	0.42	0.08	0.50	0.05
***P value***								
Mothers	0.4112		0.9342		0.3693		0.8092	
Offspring	0.1887		0.4119		< 0.0001		0.1188	
Mothers × Offspring	0.8071		0.0101		0.0452		0.0109	

The 2^−∆CT^ method was used for relative quantification of gene expression, and results are expressed as arbitrary units (AU), which represent the expression of each gene normalized to the expression of β-actin mRNA. Results are presented as mean and standard error (SE).

^a,b,c^Different superscript letters in the same column represents a significant difference by Tukey’s test (*P* < 0.05).

Maternal heat stress increased *GSS* expression in chicks grown under thermoneutral conditions. Chicks kept in a thermoneutral environment during the growth phase but hatched from heat-stressed mothers had *GSS* gene expression levels similar to those of chicks subjected to heat stress.

Offspring temperature had no effect on *HSP70* expression for chicks hatched from non-stressed mothers. However, for those hatched from heat-stressed mothers, heat stress resulted in higher *HSP70* expression than thermoneutral conditions. *MSRA* expression results followed the opposite pattern: there was no effect of offspring temperature for chicks hatched from heat-stressed mothers, but chicks hatched from non-stressed mothers and subjected to heat stress had higher *MSRA* expression compared to the chicks in thermoneutral temperature. There were no treatment effects on *GPX7* gene expression (*P* > 0.05).

Positive correlations were observed between the expression levels of *GPX7* and *HSP70* (0.66; *P* = 0.0020), *GPX7* and *MSRA* (0.70; *P* = 0.0007), and *GSS* and *HSP70* (0.61; *P* = 0.0047) in chicks (Table 6). For the mothers, however, no treatment effects were observed.

**Table 6:** Pearson correlation coefficients between the expression of glutathione peroxidase 7 (*GPX7*), glutathione synthetase (*GSS*), heat-shock protein 70 kDa (*HSP70*), and methionine sulfoxide reductase A (*MSRA*) genes in quail mothers (M) and their offspring (O).

	*GPX7*_(M)_	*GPX7*_(O)_	*GSS*_*(*M*)*_	*GSS*_(O)_	*HSP70*_(M)_	*HSP70*_(O)_	*MSRA*_(M)_	*MSRA*_(O)_
*GPX7*_(M)_	1	− 0.06	0.24	0.17	0.17	0.05	0.14	− 0.06
*GPX7*_(O)_		1	− 0.01	0.24	0.03	0.66*	− 0.10	0.70*
*GSS*_(M)_			1	0.20	0.05	0.23	0.60*	− 0.18
*GSS*_(O)_				1	0.39	0.61*	0.18	0.10
*HSP70*_(M)_					1	0.10	0.27	0.16
*HSP70*_(O)_						1	− 0.23	0.33
*MSRA*_(M)_							1	0.03
*MSRA*_(O)_								1

An asterisk (∗) indicates a significant correlation (*P* < 0.05).

## Discussion

Temperatures above thermal comfort limits are known to cause several deleterious effects on bird metabolism and performance. In laying hens, heat stress reduces egg production, egg weight, and shell thickness^17^. A previous study reported that hens subjected to heat stress had significantly lower total white blood cell count and antibody production as well as significantly higher mortality rates^18^. The negative effects of heat stress on bird performance can be attributed to different factors, such as reduced feed intake, protein synthesis, and antioxidant capacity; endocrine dysfunction; and derangement of calcium and phosphorous balance^19^. Such effects may also extend to offspring via intergenerational or transgenerational mechanisms, which have been hypothesized to allow individuals to cope better with predictable environmental fluctuations, thereby facilitating adaptation to changing environments^12^.To better understand how these mechanisms occur in birds, in the present study, we hypothesized that heat stress in the parental generation could influence heat stress coping capacities in their offspring. Chicks hatched from heat-stressed or non-stressed quail mothers were kept under thermoneutral conditions or subjected to heat stress during the growth phase; their performance, physiological indicators and oxidative metabolism were assessed during the growth phase.

The temperature selected for inducing heat stress has been previously reported to affect bird metabolism^20,21^. As expected, in the current study, high ambient temperature reduced the egg laying performance of quail mothers. Our results are similar to those of El-Tarabany et al.^2^, who investigated the performance of quail layers exposed to heat stress. The authors found that heat-stressed quail had lower feed intake, egg production, and egg mass. The complex mechanisms by which heat stress affects egg laying performance and quality are not yet fully understood, but it is known that some factors, such as low feed intake, reduce ovarian activity^22^. According to Song et al.^23^, heat stress plays an important role in the regulation of neuropeptides related to appetite in birds, such as ghrelin and cholecystokinin.

Low ovarian activity and egg production may also stem from alterations in neurotransmitter activity^24^, reduced blood flow to the ovary, and lower secretion of the hormones triiodothyronine, estrogen, progesterone, follicle-stimulating hormone, and luteinizing hormone^25^. Rozenboim et al.^26^ suggested that heat stress directly influences ovarian activity by reducing plasma levels of steroid hormones and expression of steroidogenic enzymes in follicles, which may prevent follicle development^27–29^. Pu et al.^30^ observed that the liver damage and abnormal lipid metabolism induced by high temperatures affect steroid metabolism, resulting in ovarian dysfunction associated with a decline of hierarchical follicle number and weight, egg number and weight, and chick birth weight.

Heat stress also compromises the antioxidant system of quail hens by decreasing the total antioxidant capacity of the liver. Zhang et al.^31^ found that birds under heat stress had lower glutathione levels and superoxide dismutase gene expression. As a result, heat-stressed birds showed reduced total antioxidant capacity and increased malondialdehyde content, affecting the ability to inhibit ROS^32^. In our study, the significantly lower total antioxidant capacity in heat-stressed mothers might have been due to *MSRA* downregulation. The gene encodes the protein methionine sulfoxide reductase A (MsrA), which acts by reducing oxidized methionine residues and eliminating ROS. Bin et al.^33^ demonstrated the ability of MsrA to mitigate oxidative damage.

In addition to the deleterious effects of heat stress on the mother's metabolism, the maternal heat stress has been shown to be responsible for metabolic alterations that impair embryo development, hatchability, and chick performance^34^. According to Ayo et al.^22^, low hatchability may be associated with hormonal disorder or reduced sperm penetration as a result of higher body temperature, promoting embryonic mortality. Maternal plasma corticosterone levels, which also increase under heat stress^5^, can influence reproductive hormone concentrations in the yolk. Thus, stressful situations experienced by mothers may affect offspring by reducing the amounts of reproductive hormones in the egg and the availability of nutrients for the embryo^35^. Navara and Pinson^36^ tested the hypothesis that high stress responsiveness to stressors (such as those caused by routine handling procedures) may lead to chronic accumulation and, consequently, higher corticosterone concentrations in egg yolk. As predicted, the authors observed that yolk corticosterone concentrations were significantly higher in birds with heightened response to stress and suggested that offspring hatched from stressed mothers are exposed to significantly higher corticosterone levels from egg yolk, which could permanently imprint offspring physiology and behavior.

On the other hand, maternal environment could be used as an interesting strategy to mitigate the negative effects of stress on offspring production by modifying their ability to respond to stressful conditions. There is evidence that prenatal stress may influence the response to future adverse stimuli^12^. Zulkifli et al.^37^ assessed the effect of food restriction during the first days of life on the response of birds to heat stress at 35 days of age. The authors observed that preconditioned birds had better responses such as lower H/L ratios than non-preconditioned birds. They argued that there is no need of using the same conditions during the preconditioning period and future challenges. The results, however, do not show clear evidence that the combination of factors applied in the early days of life contributed to physiological responses to later stress conditions^37^. Zimmer et al.^12^ assessed the effects of pre- and postnatal stress on three generations of quail. The authors showed that prenatal experiences affected not only the phenotype but also the pattern of neuroendocrine, physiological, and behavioral responses of offspring to stress. Videla et al.^16^ used a different strategy to prepare offspring for their future environment. The study addressed whether feed supplementation with thymol can positively modulate the immune response of adult Japanese quail when exposed to environmental challenges and influence immune variables of their eggs and offspring. Their results suggest that supplementation of adults with thymol had transgenerational effects on offspring.

As described above, early-life stress can be associated with both costs and benefits^12^. Adverse conditions experienced by the maternal generation have been shown to influence the susceptibility of offspring to challenges and diseases^38,39^. According to Dixon et al.^15^, the prenatal environment may influence offspring development. Maternal stress can produce epigenetic changes that are passed on to the progeny, altering their phenotype. Maternal exposure to stress conditions could, therefore, enhance or impair the response of subsequent generations to environmental challenges^12^. Because physiological responses of offspring can be shaped by epigenetic inheritance^12^, in our study it was expected that the offspring of heat-stressed mothers grown under heat stress conditions would also show low performance. We observed that there were no effects of maternal environment on egg weight, hatching rate, hatchability of total and fertile eggs, or chick performance at early life stages. However, these results might have been affected by the selection carried out during the experiment. Of the 30 quail mothers subjected to heat stress, only eggs from the six mothers with the best egg laying performance were collected and assessed. Although this procedure might have influenced the effects of heat stress, it was adopted to ensure that each quail hen produced at least two live chicks for further evaluation of maternal environmental effects on chick development and performance. However, despite the lack of effect of maternal heat stress on chick performance at early life stages, we observed that maternal heat stress led to lower weight gain and lower total antioxidant capacity during the growth phase, suggesting that the deleterious effects of maternal environment can be long lasting. Chicks hatched from heat-stressed mothers and exposed to heat stress during growth showed higher carbonylated protein content and *HSP70* gene expression than chicks born to heat-stressed mothers but grown under thermoneutral conditions. Maternal heat stress was also responsible for increasing *GSS* expression in chicks grown under thermoneutral conditions. Furthermore, no differences in *MSRA* gene expression were observed between chicks from heat-stressed mothers grown under thermoneutral or heat stress conditions. In line with the important role of these genes in mitigating oxidative damage, we found that chicks hatched from heat-stressed mothers and raised under heat stress conditions during growth had higher levels of carbonylated protein. These results indicate that maternal heat stress can potentialize the adverse effects of heat stress in progeny.

Our results showed that, although maternal heat stress had no negative effects on the performance and oxidative metabolism of offspring grown under thermoneutral conditions, it was associated with higher protein oxidation in offspring exposed to heat stress during growth. These results could be due in part to alterations in the expression of genes related to antioxidant capacity.

## Methods

### Ethics statement

This experiment was approved by the Ethics Committee on Animal Use (CEUA number 2402310719) of the State University of Maringá, Brazil. All animal procedures were performed according to the approved protocol and are in accordance with relevant guidelines and regulations.

### Animals and experimental design

A completely randomized design with a 2 × 2 factorial arrangement (two maternal ambient temperatures and two offspring ambient temperatures) was used to investigate the effects of maternal environment on offspring development.

### Maternal environment

Two hundred 1-day-old female Japanese quail were reared in collective cages up to 98 days of age. During this period, bird development and egg laying rate were monitored daily. At 98 days of age, 40 female quail with a mean body weight of 157.25 g and an egg laying rate of 85% were divided and subjected to two environmental conditions: thermoneutral (23 °C and 60% relative humidity) (*n* = 10) and heat stress (continuous exposure to 32 °C^20,21^ and 60% relative humidity) (*n* = 30). During the experimental period, birds were kept in individual cages with ad libitum access to feed and water. The diet met the nutritional requirements specified by Rostagno et al.^40^, providing 2795.309 kcal/kg metabolizable energy, 19.597% crude protein, 3.151% calcium, and 0.330% available phosphorus. The experiment was carried out for 38 days. From day 21 onward, 30 male breeders matched by body weight (161.2 g mean) were placed in female cages for 1 h daily. Male quail were used only for mating and kept under thermoneutral condition throughout the entire experiment. Paternal effects were minimized by rotating the males. Male birds were fed a basal diet and had ad libitum access to feed and water throughout the experiment.

Eggs were collected daily during the last 10 days of experimentation (after 8 days of mating) to allow sufficient time for fertilization to occur. Collected eggs were identified, weighed, and stored at 23 °C. On the last day of collection, all eggs from the six hens with the best egg laying performance in each group were acclimated to ambient temperature, placed in fruit nets to separate individually hatching eggs by treatment, and transferred to an incubator (Luna 240, Chocmaster, Piraquara, Paraná, Brazil) at 37 °C and 60% relative humidity. After 19 days of incubation, unhatched eggs were opened and classified as infertile eggs or dead embryos.

### Offspring

**Figure 1:**
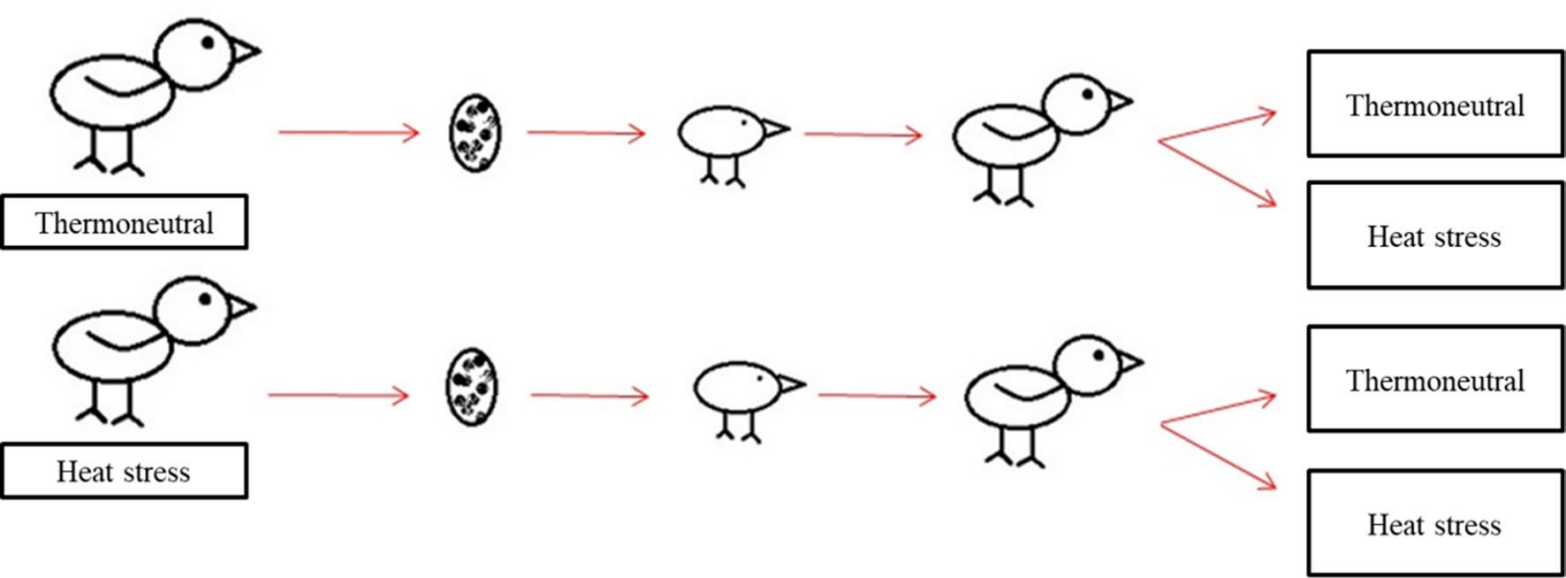
Schematic diagram of the experimental design. Eggs produced by quail layers exposed to thermoneutral and heat stress conditions were collected for 10 days and incubated under the same conditions. After hatching, chicks were raised under conventional conditions for 15 days. At 15 days of age, chicks were further divided into two groups: one half was raised under thermoneutral conditions (23 °C) and the other half under intermittent heat stress conditions (daily exposure to 34 °C for 6 h).

At hatch, all viable chicks (*n* = 140) were identified, weighed, and housed in a heated brooder according to the maternal environment group (Fig. 1). From 1 to 15 days of age, all chicks were grown under thermoneutral conditions and fed a starter diet (Table 7) with ad libitum access to water.

**Table 7:** Composition and nutrient content of starter (1 to 14 days of age) and grower (15 to 35 days of age) diets for Japanese quail (*Coturnix coturnix japonica*) chcicks.

Ingredients (%)	Phase	
	Starter	Grower
Ground corn	61.876	65.272
Soybean meal	34.000	30.600
Common salt	0.445	0.605
Soybean oil	0.300	–
Calcitic limestone	1.125	1.405
Dicalcium phosphate	1.530	1.400
*l*-Lysine HCL	0.123	0.138
*dl*-Methionine (99%)	0.185	0.165
*l*-Threonine	0.016	0.015
Vitamin–mineral premix^a^	0.400	0.400
Total	100.000	100.000
**Energy and nutrient contents**		
Apparent metabolizable energy (kcal/kg)	2898.958	2910.317
Crude protein (%)	20.694	19.395
Calcium (%)	0.855	0.924
Available phosphorus (%)	0.450	0.420
Sodium (%)	0.199	0.260
**Digestible amino acids (%)**		
Methionine + cysteine	0.750	0.701
Lysine	1.101	1.030
Threonine	0.727	0.681
Tryptophan	0.228	0.210

^a^Provided per kg of product: 2,270,000 IU vitamin A, 6330 IU vitamin E, 561 mg vitamin B1, 1490 mg vitamin B2, 858 mg vitamin B6, 3500 mcg vitamin B12, 450 mg vitamin K3, 2976 mg calcium pantothenate, 8820 mg niacin, 200 mg folic acid, 20 mg biotin, 86 mg choline, 19 mg zinc, 14 mg iron, 20 mg manganese, 3040 mg copper, 290 mg iodine, 50 mg cobalt, 88 mg selenium, 25 mg ethoxyquin, and 20 mg butylated hydroxyanisole (BHA).

At 15 days of age, female chicks were weighed and divided into two groups: thermoneutral (23 °C) and intermittent heat stress (daily exposure to 34 °C^41^ for 6 h, from 10:00 to 16:00 h). Birds in the same treatment group were housed two per cage (25 cages under thermoneutral and 25 cages under heat stress conditions, totaling 100 chicks) with ad libitum access to water and feed. From 15 to 35 days of age, all chicks were fed a grower diet formulated according to Rostagno et al.^40^.

### Evaluations

During the experimental period, quail mothers were analyzed for feed intake, feed conversion ratio (feed intake/egg mass), feed conversion ratio per dozen eggs, egg production, egg laying rate, egg weight, and egg mass, as described by Bastos et al.^42^. Hatchability, hatchability of fertile eggs (%), and embryonic mortality (%) were calculated according to Kopenol et al.^43^. Chick performance was analyzed for starter (1–14 days of age) and grower (15–35 days of age) phases.

At the end of the egg collection period, quail mothers were euthanized by cervical dislocation, and chicks were slaughtered by the same procedures at 35 days of age. Mature follicles (F1 phase) were extracted from the ovary of mothers, weighed, and counted. Relative weights of the ovary, liver, heart, intestine, and spleen were calculated by dividing the organ weight by the bird weight and multiplying by 100.

For gene expression analysis, a section of the left lobe of the liver of mothers and chicks was collected. Samples were preserved with RNAlater (Life Technologies, São Paulo, Brazil) and stored at − 20 °C until total RNA extraction. Blood samples were collected into heparinized tubes for determination of the heterophil/lymphocyte ratio. Liver specimens (right lobe) were collected in liquid nitrogen and stored at − 80 °C for biochemical analyses (total antioxidant capacity, carbonylated proteins, and lipid peroxidation).

### Gene expression

Liver samples from five chicks from each treatment were used for gene expression analysis. Total RNA was extracted from 80 mg of liver tissue using 1 mL of TRIzol (Invitrogen, Carlsbad CA, USA), according to the manufacturer’s protocol. RNA integrity was assessed by electrophoresis on 1% agarose gels, followed by ethidium bromide (10 mg/mL) staining and visualization under ultraviolet light.

RNA samples were treated with DNase I (Invitrogen, Carlsbad, CA, USA) to eliminate DNA contamination. The GoScript Reverse Transcription kit (Promega, Madison, WI, USA) was used for complementary DNA (cDNA) synthesis from 4 µL of DNase-treated RNA, following the manufacturer’s instructions.

Real-time (RT) PCR (RT-qPCR) was performed using 5 µL of cDNA diluted to 40 ng/µL, 0.5 µL of each primer diluted to 10 µM, 12.5 µL of SYBR Green PCR Master Mix (Applied Biosystems, Waltham, MA, USA), and 6.5 µL of ultrapure water in a final volume of 25 µL. A pool of cDNA samples was serially diluted (10, 20, 40, and 80 ng/µL) and used to assess the efficiency of the primers. Thermocycling conditions were the same for all genes: 95 °C for 10 min, followed by 40 cycles of 95 °C for 15 s for denaturation and 60 °C for 1 min for annealing/extension. Melting curves were obtained to assess the specificity of amplification.

Primers (Table 8) used for amplification of glutathione peroxidase 7 (*GPX7*), glutathione synthetase (*GSS*), heat-shock protein 70 kDa (*HSP70*), and methionine sulfoxide reductase A (*MSRA*) genes were designed from sequences deposited in GenBank (www.ncbi.nlm.nih.gov). The β-actin gene was used as endogenous control. PCR analyses were carried out in duplicate. Amplification efficiencies were similar for all target genes, ranging from 90 to 110%. The 2^−∆CT^ method was used for relative quantification of gene expression^44^.

**Table 8:** Primers used in RT-qPCR.

Gene^a^	bp^b^	Orientation	Sequence (5′ → 3′)	Accession number
*GPX7*	140	Forward	GGTGCCTCCTTTCCTATGTT	NM_001163245.1
		Reverse	AGTTCCAGGTTGGTTCTTCTC	
*GSS*	108	Forward	GTGCCAGTTCCAGTTTTCTTATG	XM_425692.3
		Reverse	TCCCACAGTAAAGCCAAGAG	
*HSP70*	65	Forward	ATGAGCACAAGCAGAAAGAG	NM_001006685.1
		Reverse	TCCCTGGTACAGTTTTGTGA	
*MSRA*	76	Forward	ATGACCCGACACAAGGAATG	XM_004935891
		Reverse	TGGGAAAAGGTGTAGATGGC	
β-Actin	136	Forward	ACCCCAAAGCCAACAGA	L08165.1
		Reverse	CCAGAGTCCATCACAATACC	

^a^*GPX7* glutathione peroxidase 7 gene, *GSS* glutathione synthetase gene, *HSP70* heat-shock protein 70 kDa gene, *MSRA* methionine sulfoxide reductase A gene.

^b^*bp* base pairs. The annealing temperature was 60 °C for all genes.

### Biochemical analyses

Total antioxidant capacity was measured according to the method described by Brand-Williams et al.^45^, with modifications. For this analysis, 100 mg of liver tissue was added to a test tube containing 1 mL of methyl alcohol, homogenized, and centrifuged for 10 min at 10,000×*g* and 4 °C. A 22.5 µL aliquot of the supernatant was added to a microplate containing 277.5 µL of 0.06 mM 2,2-diphenyl-1-picrylhydrazyl (DPPH, Sigma–Aldrich, St. Louis, MO, USA), in duplicate. Microplates were kept in the dark for 30 min, after which the absorbance was read at 515 nm using a microplate reader (VersaMax, Molecular Devices, San Jose, CA, USA). The antioxidant capacity of each sample was calculated as follows: Total antioxidant activity (%) = (1 − (absorbance of the sample/absorbance of DPPH)) × 100.

Lipid peroxidation was determined by the thiobarbituric acid reactive substances (TBARS) method. Briefly, 100 mg of liver tissue was added to a test tube containing 1 mL of 0.1 M potassium phosphate buffer (pH 7.4), homogenized, and centrifuged for 10 min at 10,000×*g* and 4 °C. After centrifugation, 500 µL of the supernatant was transferred to a new microtube containing 250 µL of 28% trichloroacetic acid diluted in 0.25 N hydrochloric acid, 250 mL of 1% thiobarbituric acid diluted in 1:1 acetic acid, and 125 mL of butylhydroxytoluene diluted in ethanol. The solution was homogenized, incubated at 95 °C for 15 min, and centrifuged for 10 min at 10,000×*g* and 4 °C. TBARS concentration was determined in duplicate using aliquots of 300 µL. The absorbance was read (VersaMax, Molecular Devices) at 535 nm, and results are expressed as nmol TBARS/mg protein.

Protein oxidation was estimated by quantification of carbonylated derivatives using 2,4-dinitrophenylhydrazine (DNPH, Sigma-Aldrich, Saint Louis, MO, USA), as described by Levine et al.^46^. Absorbance was read (VersaMax, Molecular Devices) at 370 nm. Carbonylated protein concentrations were determined using the Beer–Lambert equation: *A* = *C* × *b* × *ε*, where *A* is the absorbance of the sample minus that of the blank, *C* is the concentration of carbonylated proteins, *b* is the optical path length, and *ε* is the molar attenuation coefficient (22,000 mol/L cm). Results are expressed as nmol carbonylated protein/mg protein.

Total proteins were determined by the Bradford method^47^.

### Heterophil/lymphocyte (H/L) ratio

A 10 µL aliquot of blood was smeared on a microscope slide using a spreader slide until reaching the edges of the slide. Slides were left to air dry, stained using a blood staining kit (Instant Prov, Newprov, Pinhais, Paraná, Brazil), and examined under an optical microscope. A total of 100 cells (heterophils and lymphocytes) were counted to determine the H/L ratio^48^.

### Statistical analysis

#### Data analysis

Data from quail mothers were subjected to procedure of one-way analysis of variance (ANOVA), and the means were separated using *t* test. To assess the effects of the maternal environment on the performance, physiological parameters and oxidative metabolism of the offspring during the growth phase, the interaction between hen temperature and offspring temperature was analyzed as completely randomized design with a 2 × 2 factorial arrangement, using general linear model (GLM) procedure of two-way analysis of variance (SAS Inst. Inc., Cary, NC, USA). The means were separated using Tukey's multiple range test procedures when treatment effect was significant (*P* < 0.05) (SAS Inst. Inc., Cary, NC, USA). Prior to applying ANOVA, all data were tested for homogeneity of variance by Levene’s test and normal distribution by Kolmogorov–Smirnov test. Results are presented as mean and standard error (SE). Pearson correlation analysis was performed to investigate associations between gene expression in quail mothers and their offspring as well as between target genes.

## Data Availability

The datasets generated during the current study are available from the corresponding author on reasonable request.
